# From Galls to Cecidological Herbaria: The Role of Gall Collections in Modern Life Sciences

**DOI:** 10.3390/life14040452

**Published:** 2024-03-29

**Authors:** Mauro Mandrioli, Luca Tonetti, Tiziana Beltrame, Elena Canadelli

**Affiliations:** 1 Dipartimento di Scienze della Vita, Università di Modena e Reggio Emilia, Via Campi 213/D, 41125 Modena, Italy; 2 Dipartimento di Scienze Storiche, Geografiche e dell’Antichità, Università di Padova, Via del Vescovado 30, 35141 Padova, Italy; luca.tonetti@unipd.it (L.T.); tiziana.beltrame@unipd.it (T.B.); elena.canadelli@unipd.it (E.C.)

**Keywords:** galls, cecidia, gall collections, botanical collection, ecology, biodiversity

## Abstract

Galls (also known as cecidia) have been studied by botanists, zoologists and microbiologists over the last century. Indeed, galls can be induced by different animals, bacteria, viruses and fungi, so that their presence simultaneously attested the presence of specific host plants and gall-inducing species. Consequently, gall collections, also known as cecidological herbaria or cecidological collections, can be interesting to study biodiversity changes over time. This review describes the main cecidological collections currently available in different European museums in order to stimulate their future study. The present analysis suggests that well-organized and preserved cecidological collections have great potential to guide research in taxonomy and systematics. Furthermore, this review aims to encourage future research on the conservation and digitisation standards of gall specimens in order to make cecidological data more accessible to researchers.

## 1. Cecidological Herbaria as Unusual Museum Collections in Germany and Italy

**Figure 1:**
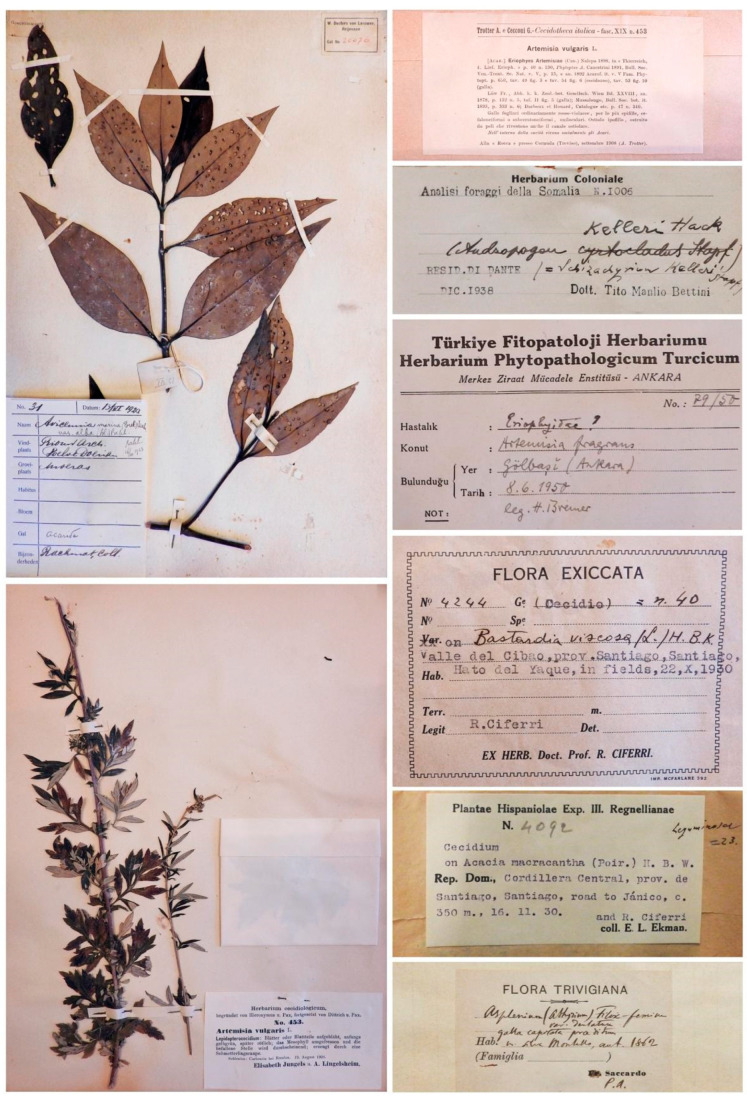
Examples of cecidological herbaria collected in the late 19th and early 20th centuries are shown in the figure. These herbaria are among the most studied worldwide for examining gall morphology and distribution in different countries and habitats. The specimens in the photographs are preserved at the Botanical Museum of the University of Padua, Italy.

At the end of the nineteenth century, several naturalists (mainly botanists) focussed their attention on galls by sampling them and creating collections, usually called cecidological herbaria [1,2,3]. Even though galls were often sampled from the same or similar host plant species, cecidological herbaria (Figure 1) may be differently organised. Indeed, galls may be enclosed in envelopes, collected in standard herbarium sheets or included in mixed collections consisting of envelopes, sheets and boxes containing galls and/or the gall-inducing species [1,2,3,4,5,6].

Envelopes, even if not typically used in plant herbarium collections [7], have been frequently utilized for storing cecidia, especially galls on leaves or large galls. Folded-paper envelopes are found in many cecidological herbaria as they make it easier to observe and analyse specimens [4,5,6]. However, a drawback of using envelopes is that they need to be opened to view the specimens. This method of gall conservation is popular among botanists involved in mycology and bryology. Transparent envelopes, like those used for Odonata collections, provide better visibility and minimize handling, but they are not widely used [1,2].

Herbarium sheets require specialized cabinets, which are not typically found in gall collections. The non-uniform size of galls presents a challenge for their conservation as standard plant herbarium samples. Only flatter types of galls or small-sized samples are suitable for herbarium sheets, as thick, heavy, and protruding galls can cause problems with storage. Despite these issues, herbarium sheets offer excellent visibility of galls, making them the preferred method for plant gall conservation [1,2,3,4,5,6].

In the first half of the 20th century, cecidological studies were very common in Germany, and several naturalists, in particular botanists, collected galls that were successively included in internationally exchanged cecidological herbaria [8]. Among these collections, a well-known cecidological herbarium was the *Otto Jaap*—*Zoocecidien-Sammlung* started in 1910 by botanist Otto Jaap (1864–1922), who built up a large collection of galls consisting of 846 specimens [2,9]. The collection was arranged in 34 series, with each specimen accompanied by data about the host plant, the date and place of sampling and the taxonomic description of the gall-inducing species [2,9].

After his retirement at the age of 48 from his role as school professor, Jaap published several studies on galls collected in numerous locations in Germany (and in particular in Brandenburg and Hamburg), Dalmatia and Istria [2]. His original specimens are now preserved in the Ross’s cecidological herbaria at the *Botanische Staatssammlung München* that is part of the Bavarian Natural History Collections [8,9]. Copies of the Otto Jaap’s plant gall collection, consisting of galls collected within folded-paper envelopes, can be found in several European botanical and natural history museums [1,8].

The *Herbarium Cecidologicum*, curated by Hans Emmo Wolfang Hieronymus (1846–1921), a botanist specialised in ferns and algae, was a very popular gall collection in Germany [8]. Hieronymus collected galls for several years in various countries, with most of his work conducted while serving as a curator at the *Botanischer Garten und Botanisches Museum Berlin-Dahlem and Botanisches Museum Universität Breslau* in Wroclaw, Poland [8,9]. Upon Hieronymus’s death, botanist Ferdinand Albin Pax (1858–1942) sampled additional specimens to enrich the cecidological collection [3,8]. Together, Hieronymus and Pax provided the most comprehensive overview of plant galls in Germany during their time. The interest in cecidology in Germany at the end of the 1800s is also evident from the presence of two volumes of the *Hieronymus und Pax Herbarium Cecidologicum* at the German exhibit of the World’s Columbian Exposition, held in Chicago in 1893, showcasing significant botanical discoveries. The exhibit featured two volumes displaying German hymenopterous galls, phytopterous galls and dipterous galls [10]. The *Hieronymus und Pax Herbarium Cecidologicum* contained 691 specimens and the original specimens are now preserved at the *Botanische Staatssammlung München* [8].

Gall collections were also common in Italy, such as the *Erbario Cecidologico* at the botanical section of the Museum of Natural History of the University of Florence [4]. A similar cecidological herbarium, also called *Erbario Cecidologico*, is part of the botanical collections at the University of Ferrara. It contains 575 Italian galls, together with specimens from Tripoli, organized in 23 folders. All specimens are well-preserved and intact providing information on location, date of collection of the plant, and taxonomy of the gall-inducing species, primarily insects [11].

The Museum of Natural History at the University of Florence also possesses a copy of the cecidological herbarium assembled between 1900 and 1918 by the botanist Alessandro Trotter (1874–1967) and the entomologist Giacomo Cecconi (1866–1941), known as *Cecidotheca Italica*. This collection consists of 575 specimens organized in 23 folders [4]. Trotter and Cecconi preserved their galls in paper envelopes that were mounted on paper sheets. Each specimen included a fragment of the host plant and a printed label with the names of the host plant and the gall inducer and the place and date of collection [5]. This collection has been extensively studied for its scientific and historical significance, serving as a valuable resource for naturalists interested in cecidology, from both botanical and zoological perspectives [4]. Copies of the *Cecidotheca Italica* are now held in various institutes and universities in Italy, such as the Herbarium Museum of the University of Cagliari and the Botanical Museum of the University of Padua [12].

In the early 20th century, gall collections, such as the *Cecidotheca Italica*, were widely used in Italy for studying the biodiversity of galls in a given geographical area and for educational purposes. Interestingly, the Alessandro Trotter Collection, present at the Botanical Museum of the University of Padua since 1952, includes not only Trotter’s own gall specimens, but also samples from other international cecidological herbaria that were either acquired or donated to Trotter.

Trotter was regarded as the foremost cecidologist in Italy [13,14,15] and in 1902 he founded *Marcellia. Rivista internazionale di cecidologia*, an international scientific journal dedicated to cecidology, with a particular focus on the biology and systematics of galls. The title of the journal was inspired by Marcello Malpighi (1628–1694), an Italian physician who is considered the ‘father’ of cecidology [15,16,17,18,19,20]. Trotter’s personal collection of galls is noteworthy since it consists of more than 5000 specimens including samples from various other gall collectors, such as Jaap and Pax, and samples collected and/or studied by Trotter himself [16,17,18,19]. In 1952, the Trotter collection was donated to the University of Padua and it has been organized into 43 folders, alphabetically arranged according to the plant scientific names [12,21]. The donation also included his private archive and library that are now preserved in the *Biblioteca storica di medicina e botanica Vincenzo Pinali e Giovanni Marsili* at the University of Padua. An interdisciplinary project is currently undergoing at the Botanical Museum of Padua to study the Trotter collection. The project focuses on two main aspects: first, the historical reconstruction of Trotter’s role in botany at the beginning of the 20th century; and second, the inventory and revision of specimens in his gall collection. The Filippo Silvestri Entomological Museum, which is affiliated with the former Department of Entomology and Agricultural Zoology at Federico II University of Naples, also houses a cecidological collection attributed to Trotter that will be studied in the near future. This collection comprises approximately 4000 specimens, including 575 specimens from the *Cecidotheca Italica* [22].

## 2. An International Interest for Galls: Cecidological Collections in Middle and North Europe

The interest in gall study in the first half of the 20th century is attested by the presence of cecidological collections in several European countries. One notable collection is preserved at the Botanic Garden in Meise, Belgium, where over 2500 herbarium sheets were deposited by Belgian botanists Joseph-Edgard De Langhe (1907–1998) and Jacques Larnbinon (1936–2015) [23]. Although the collection does not have a specific reference to cecidology in its name, it predominantly consists of plant galls, including rare specimens collected by De Langhe and identified by Willem Marius Docters Van Leeuwen (1880–1960), a Dutch botanist and entomologist, known for his work on insect–plant interactions, including galls [24,25]. Docters van Leeuwen, along with his wife Jenny Docters van Leeuwen-Reijnvaan (1880–1963), published several papers and books on plant galls, such as the notable book *The Zoocecidia of the Netherlands East Indies*, featuring over 1100 ink drawings of galls by the Javanese artist Raden Sastrasaputra [25,26]. De Langhe and Docters van Leeuwen also collaborated with the Italian botanist Cecconi [26], whose collection of galls is now part of the Trotter collection at the Botanical Museum of the University of Padua, Italy.

**Figure 2:**
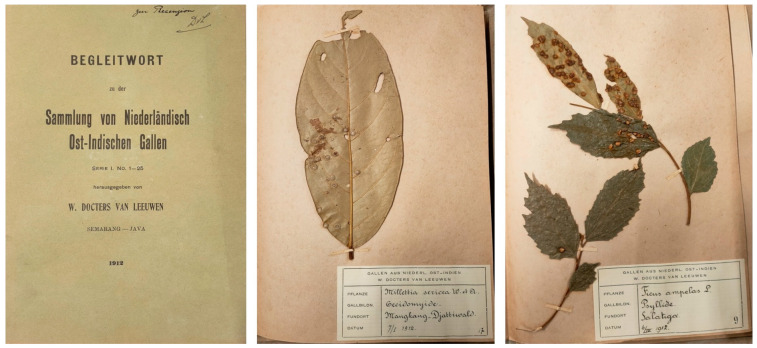
Cover and two specimens from the *Sammlung Nied. Ost-Indischer Gallen* collected by Willem Marius Docters van Leeuwen in 1912. The photographed specimens are preserved at the Botanical Museum of the University of Padua, Italy.

As confirmed in the first *Supplement* to *The Zoocecidia of Netherlands’ East Indies* [24,25], the study of gall biology was a significant focus of Willem Marius Docters van Leeuwen and Jenny Docters van Leeuwen-Reijnvaan during their permanence in the ‘Dutch East Indies’ (specifically, Central Java) from January 1908 to August 1932. Indeed, since 1912 Docters van Leeuwen compiled and published catalogues of galls from Java and other parts of the Malay Archipelago. Each folder contained 25 sheets of 18 × 28 cm, with specimens directly pinned to the sheet (Figure 2). A handwritten label provided information on the host plant, the gall-inducing organism, and the location/date of collection. The introductory booklet included a description of all samples with bibliographical references, drawings, and some photographs [27].

**Figure 3:**
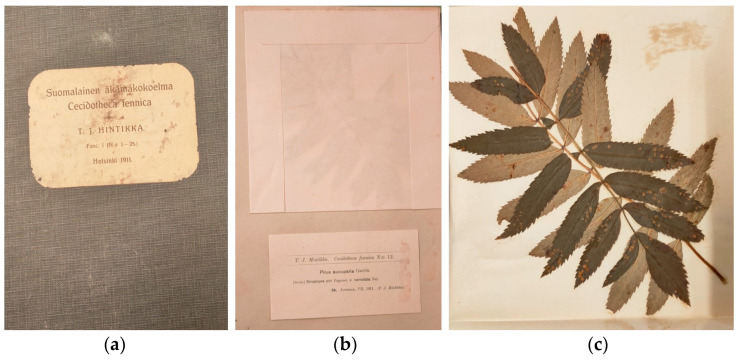
Cover of folder 1 (**a**) and specimen no. 12, (**b**) inside the envelope and (**c**) removed from the envelope, from the *Cecidotheca Fennica* compiled by Toivo Juho Hintikka between 1911 and 1913. The photographed specimens are preserved at the Botanical Museum of the University of Padua, Italy.

The *Cecidotheca Fennica* (or *Suomalainen äkämäkokoelma*) was compiled by the Finnish botanist Toivo Juho Hintikka (1888–1952) between 1911 and 1913. This collection, consisting of only 50 specimens organized in two folders, is of great interest as it provides a unique representation of the biodiversity of galls in Finland. Each gall is enclosed in a special paper envelope accompanied by a numbered label detailing the plant host, gall-inducing species, and sampling location (Figure 3). The folders are provided with two alphabetical indexes: one for host plants and the other for gall-inducing organisms. Galls are arranged systematically according to host plant taxonomy. In addition to his work on plant pathology, Hintikka is also renowned for his first revision of Finnish Myxomycetes and his exsiccate collection of 20 species of Myxomycetes (*Myxogastres fennici exsiccati*) [28].

**Figure 4:**
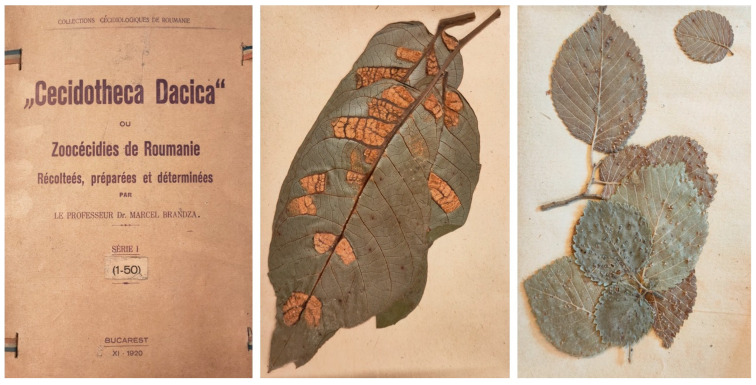
Cover and two specimens from the *Cecidotheca Dacica* compiled by Marcel Brândză. The photographed specimens are preserved at the Botanical Museum of the University of Padua, Italy.

Differently from the previously described gall collections, the *Cecidotheca Dacica*, a zoocecidia herbarium compiled by the Romanian botanist Marcel Brândză (1868–1934), is organized by gall inducers rather than by host plant [29]. This herbarium, which is considered the first cecidological reference collection in Romania, consists of approximately 300 sheets, with specimens arranged in paper envelopes. Each sheet shows the inscription “Dr. M. Brândză, Cecidotheca Dacica” (Figure 4). Occasionally, specimens are marked as “Houard, Zoocecid. No…”, indicating the rank number of the species identified in Clodomir Houard’s catalogue [6], which Brândză used to verify his collection of gall-inducing species. Labels, in French, provided the name of the gall inducer, host plant, and place/date of collection. Samples were collected and identified by Brândză between November 1920 (series I, II, III, and IV) and April 1921 (series V and VI) [29].

**Figure 5:**
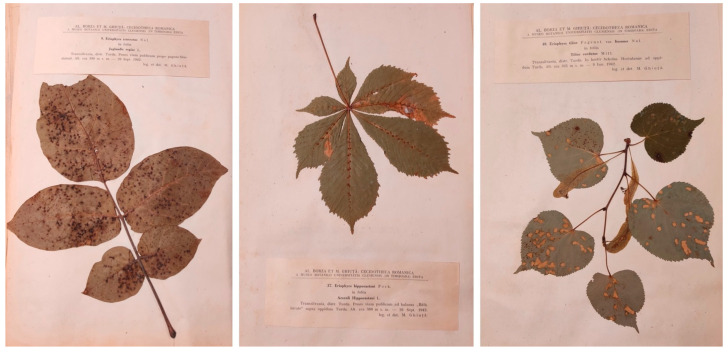
Examples of specimens from the *Cecidotheca Romanica* collected by Alexandru Borza. The photographed specimens are preserved at the Botanical Museum of the University of Padua.

Supported by Mihai Ghiuță, the botanist Alexandru Borza (1887–1971) established the *Cecidotheca Romanica*, another Romanian gall collection consisting of approximately 200 samples organized in 4 series [30,31]. Labels are written in Latin and primarily report the name of the gall-inducing organism, along with the location, date of the collection, and sometimes the altitude (Figure 5). Borza might have been influenced by the German cecidological collections, which he had the opportunity to study during his PhD. During this time, he met German botanists Adolf Engler Pax (1844–1930) and Oskar Eberhard Ulbrich (1879–1952). Borza made significant contributions to Romanian botany and his gall collection is preserved at the Botanical Garden of the University of Cluj (now known as Alexandru Borza Cluj-Napoca University Botanic Garden), which he reorganized in the early 1920s and directed from 1919 to 1947 [30,31].

**Figure 6:**
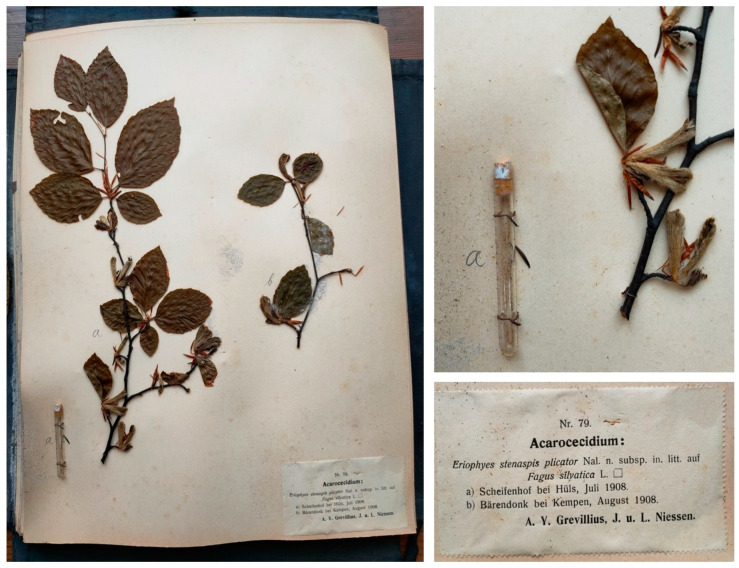
The *Zoocecidia et Cecidozoa* collection, compiled by Anders Yngve Grevillius and Josef Niessen, consists of separate specimens corresponding to the plant host (fixed on paper sheets) and the gall-forming organisms stored in vials. The photographed specimens are preserved at the Botanical Museum of the University of Padua, Italy.

A further noteworthy example of gall collection is the *Zoocecidia et Cecidozoa* collection (6 series, 150 specimens), compiled and published from 1906 to 1912 in Cologne by botanists Anders Yngve Grevillius (1864–1925) and Josef Niessen (1864–1942). Galls and their inducing organisms were collected separately and stored in vials and on paper sheets, respectively. Each specimen was labelled with species name, host plant, location, and date (Figure 6). The collection includes an index and a booklet with detailed descriptions of each gall. The preservation of this extensive and delicate collection is challenging due to the vials and plant specimens.

Various natural history museums worldwide, such as the Natural History Museum in London (see https://lnhsgallcollection.myspecies.info/gallery?page=1, accessed on 19 March 2024), possess cecidological herbaria. However, many of these collections are currently understudied, hindering the assessment of their conservation status. Prioritizing the description of these collections is crucial for a better understanding and conservation of the biodiversity they represent. An example of this is the ongoing reconditioning project for the collection of mites sampled by Austrian acarologist Alfred Nalepa (1856–1929) [32], which is expected to yield valuable new data in the near future.

## 3. Future Perspective in the Study of Gall Collections

Cecidological collections represent a unique challenge for natural history museums due to the lack of uniform curatorial guidelines for their conservation and digitisation, unlike the well-established standards for plant herbaria [1].

The State Museum of Natural History in Stuttgart has recently examined historical methods of gall preservation and explored how digitization of specimens can be planned and managed [1]. In particular, Mertz and colleagues [1] suggest an organization of cecidological collections by host plants to facilitate research at the ecological community level diverging from the traditional organization by the taxonomy of the gall inducers. This proposal is similar to what is found in several historical cecidological collections (such as the *Cecidotheca Italica* by Trotter and Cecconi), but differs from the organization of gall collections in several Botanical Museums, where galls are categorized by inducers such as bacteria, fungi and animal species, with a further distinction between zoocecidia induced by mites and insects [1,8].

At the same time, the analysis by Mertz and colleagues [1] highlights the challenges faced by museum curators in managing gall collections due to changes in taxonomic nomenclature of plants and gall-inducing species over the past two centuries. Thus, historical revision of specimen labels is often necessary. Digitizing gall collections may be useful to generate biodiversity maps for plants and gall-inducing species, also identifying underexplored areas of gall diversity [1]. Indeed, in view of their biological origin [33,34,35], galls are of interest in both botany and zoology, as they concern both the plants that are attacked and the organisms that induce galls. At the same time, since each gall represents a specific response of plants to the gall-inducing species [36,37,38,39,40], the study of galls may provide insights into plant–insect interactions, enhancing our understanding of the complex relationships occurring among organisms in ecosystems [41,42,43,44,45]. Moreover, since the formation of galls is triggered by sophisticated molecular mechanisms orchestrated by plants, gall collections may shed light on the evolutionary strategies employed by both plants and gall-inducing species highlighting their interdependent relationship [46,47,48,49,50].

Unfortunately, the available cecidological collections are not focussed on specific gall inducer taxa, but they mainly represent the distribution of galls in different countries and/or on different host plants. Furthermore, the current content of these collections is often poorly documented as cecidological herbaria have not been extensively studied in recent decades. This review of the main gall collections available in Europe aims to generate interest among scientists in life sciences in order to promote the digitisation and study of these collections. At this regard, several digitisation projects of herbaria are currently ongoing in different countries, such as Italy and Germany, where different pipelines and tools have been successfully employed and can be adapted for digitizing gall collections as well [7].

The availability of gall collections can support new sampling projects for comparing present and past biodiversity. This is particularly relevant in countries like Italy, where national projects are underway to study biodiversity [7]. For instance, the interest for the Italian natural history collections has been greatly stimulated in the last two years by the establishment of the *National Biodiversity Future Center* (NBFC, www.nbfc.it/, accessed on 22 February 2024). NBFC aims at conserving, restoring, monitoring and enhancing Italian and Mediterranean biodiversity [7]. One of its key activities is the large-scale digitization of the *Herbarium Centrale Italicum* in Florence (Italy), following international standards for data accessibility and interoperability [7]. This large-scale digitization project will improve accessibility to herbaria and enable high-throughput workflows that can be applied to other Italian plant collections, including cecidological ones. The digitisation of gall collections will make data on gall-inducing species available to researchers through aggregated portals, such as *Global Biodiversity Information Facility* (GBIF, https://www.gbif.org, accessed on 19 February 2023) and *iDigBio* (https://www.idigbio.org/, accessed on 19 February 2023).

Cecidological collections are also extremely fascinating from an historical point of view since the debate about the mechanism of gall induction has been recurrent throughout the history of science. Indeed, galls have been studied by several naturalists, such as Ulisse Aldrovandi (1522–1605), Francesco Redi (1626–1679), Marcello Malpighi and Antonio Vallisneri (1661–1730) [51,52,53,54,55,56]. These studies were revived at the end of the 19th century with the establishment of cecidology as a scientific discipline. This was also due to the applicability of such studies in agriculture and industry. Thus, today cecidological herbaria are invaluable resources also for historians of science.

A well-organized gall collection has therefore the great potential to guide research in taxonomy and systematics, as well as in ecology. This scientific interest should prompt future research aimed at standardising gall specimen conservation and digitisation in order to have publicly available cecidological data to researchers involved not only in the study of gall biology, but also in both animal and plant taxonomy, and history of science.

## Data Availability

Not applicable.

## References

[1] Mertz A.K., Awad J., Wendt I., Dalitz C., Krogmann L. (2022). Curation and digitization of insect galls in the collection of the State Museum of Natural History Stuttgart. Integr. Syst..

[2] Ross H. (1922). Otto Jaap. Z. Pflanzenkrankh. Gallenkd..

[3] Rat M., Anačkov G. (2012). Herbarium BUNS: Plant gall collection. Proc. Nat. Sci. Matica Srp..

[4] Moggi G., Raffaeli M. (2009). The gall and teratological herbaria. The Museum of Natural History of the University of Florence: The Botanical Collections.

[5] Trotter A., Cecconi G. (1900–1917). Cecidotheca Italica o Raccolta di Galle Italiane Determinate, Preparate ed Illustrate.

[6] Houard C. (1908–1913). Les Zoocécidies des Plantes d’Europe et du Bassin de la Méditerranée.

[7] Mandrioli M. (2023). From dormant collections to repositories for the study of habitat changes: The importance of herbaria in modern life sciences. Life.

[8] Skuhravá M., Skuhravý V., Meyer H. (2014). Gall midges (Diptera: Cecidomyiidae: Cecidomyiinae) of Germany. Faun.-Okol. Mitt..

[9] Roessler H., Hrtel H., Schreiber A. (1988). Gallen-Herbar. Die Botanische Staatssammlung München 1813–1988. Eine Übersicht über die Sammlungsbestände.

[10] (1893). Botany at the World’s Fair. Bot. Gaz..

[11] Baldi C., Iannucci P. (2021). Storie di libri e di palazzi. Alla Scoperta del Patrimonio Culturale dell’Università di Ferrara.

[12] Pellizzari G., Minelli A. (1995). La cecidoteca. L’Orto botanico di Padova 1545–1995.

[13] Di Stefano M. (1967). Lineamenti cecidologici di un maestro. Marcellia.

[14] De Natale A., Pollio A. (2012). A forgotten collection: The Libyan ethnobotanical exhibits (1912–14) by A. Trotter at the Museum O. Comes at the University Federico II in Naples, Italy. J. Ethnobiol. Ethnomed..

[15] Ottaviani A. (2020). Trotter, Alessandro. Dizionario Biografico Degli Italiani.

[16] Massalongo C. (1898). Le galle nell’*Anatome plantarum* di M. Malpighi. Malpighia.

[17] Trotter A. (1902). Al lettore. Marcellia.

[18] Trotter A. (1902). Progresso e importanza degli studi cecidologici. Marcellia.

[19] Trotter A. (1928). La Cecidologia. Nostre conoscenze intorno alle galle. Rivista di Fisica, Matematica e Scienze Naturali.

[20] Di Stefano M. (1968). Elenco completo delle monografie e degli studi Cecidologici del Trotter. Marcellia.

[21] Tornadore N., Gregolin C. (1996). Le Collezioni botaniche. I Musei, le Collezioni Scientifiche e le Sezioni Antiche Delle Biblioteche.

[22] De Natale A., Mazzoleni S., Pignatelli S. (2007). Herbarium Porticense. I Musei Delle Scienze Agrarie. L’evoluzione Della Wunderkammer.

[23] Carbonnelle S. (2018). Hidden secrets in boxes: Belgian plant galls collections reveal secrets. Cecidology.

[24] Docters van Leeuwen W.M. (2009). Gallenboek. Overzicht van Nederlands Gallen (Neuauflage).

[25] Docters van Leeuwen-Reijnvaan J., Docters van Leeuwen W.M. (1941). The Zoocecidia of the Netherlands’ East Indies. Supplement I. Ned. Kruidkd. Arch..

[26] Kolesik P., Gagnè R.J. (2020). A review of the gall midges (Diptera: Cecidomyiidae) of Indonesia: Taxonomy, biology and adult key to genera. Zootaxa.

[27] Docters van Leeuwen W.M. (1912). Begleitwort zu der Sammlung von Niederländisch Ost-Indischen Gallen, Serie I. No. 1–25.

[28] Härkönen M. (1978). A check-list of Finnish myxomycetes. Karstenia.

[29] Stănescu M. (2009). The Catalogue of the “Dr. Marcel Brândză” Zoocecidia Herbarium from the “Grigore Antipa” National Museum of Natural History (Bucureşti). Trav. Mus. Natl. d’Histoire Nat. ‘Grigore Antipà.

[30] Vonica G. (2006). Herbarul Alexandru Borza din colecţiile Muzeului de Istorie Naturală Sibiu. Acta Musei Maramorosiensis.

[31] Ciobanu D.G., Sinitean A. (2018). The titan of Romanian botany: Alexandru Borza. Biostudent.

[32] Chetverikov P., Hörweg C., Kozlov M., Amrine J. (2016). Reconditioning of the Nalepa collection of eriophyoid mites (Acariformes, Eriophyoidea). Syst. Appl. Acarol..

[33] Shorthouse J.D., Wool D., Raman A. (2005). Gall-inducing insects. Nature’s most sophisticated herbivores. Basic Appl. Ecol..

[34] Stireman J.O., Devlin H., Carr T.G., Abbot P. (2010). Evolutionary diversification of the gall midge genus *Asteromyia* (Cecidomyiidae) in a multitrophic ecological context. Mol. Phylogenet. Evol..

[35] Joy J.B., Crespi B.J. (2007). Adaptive radiation of gall-inducing insects within a single host-plant species. Evolution.

[36] Stone G.N., Schönrogge K. (2003). The adaptive significance of insect gall morphology. Trends Ecol. Evol..

[37] Trotter A. (1899–1900). I micromiceti delle galle. Atti Ist. Ven..

[38] Takeda S., Hirano T., Ohshima I., Sato M.H. (2021). Recent progress regarding the molecular aspects of insect gall formation. Int. J. Mol. Sci..

[39] Raman A., Schaefer C.W., Withers T.M., Raman A., Schaefer C.W., Withers T.M. (2005). Galls and gall-inducing arthropods: An overview of their biology, ecology, and evolution. Biology, Ecology, and Evolution of Gall-Inducing Arthropods.

[40] Espírito-Santo M.M., Fernandes G.W. (2007). How many species of gall- inducing insects are there on earth, and where are they?. Ann. Entomol. Soc. Amer..

[41] Giannetti D., Mandrioli M., Schifani E., Castracani C., Spotti F.A., Mori A., Grasso D.A. (2021). First report on the acrobat ant *Crematogaster scutellaris* storing live aphids in oak-gall nests. Insects.

[42] Dreger-Jauffret F., Shorthouse J.D., Shorthouse J.D., Rohfritsch O. (1992). Diversity of gall-inducing insects and their galls. Biology of Insect-Induced Galls.

[43] Giron D., Huguet E., Stone G.N., Body M. (2016). Insect-induced effects on plants and possible effectors used by galling and leaf-mining insects to manipulate their host-plant. J. Insect Physiol..

[44] Mani M.S. (1964). Ecology of Plant Galls.

[45] Gätjens-Boniche O. (2019). The mechanism of plant gall induction by insects: Revealing clues, facts, and consequences in a cross-kingdom complex interaction. Rev. Biol. Trop..

[46] Takeda S., Yoza M., Amano T., Ohshima I., Hirano T., Sato M.H., Sakamoto T., Kimura S. (2019). Comparative transcriptome analysis of galls from four different host plants suggests the molecular mechanism of gall development. PLoS ONE.

[47] Zhao C., Escalante L.N., Chen H., Benatti T.R., Qu J., Chellapilla S., Waterhouse R.M., Wheeler D., Andersson M.N., Bao R. (2015). A massive expansion of effector genes underlies gall formation in the wheat pest *Mayetiola destructor*. Curr. Biol..

[48] Korgaonkar A., Han C., Lemire A.L., Siwanowicz I., Bennouna D., Kopec R.E., Andolfatto P., Shigenobu S., Stern D.L. (2021). A novel family of secreted insect proteins linked to plant gall development. Curr. Biol..

[49] Schultz J.C., Edger P.P., Body M.J.A., Appel H.M. (2019). A galling insect activates plant reproductive programs during gall development. Sci. Rep..

[50] Bartlett L., Connor E.F. (2014). Exogenous phytohormones and the induction of plant galls by insects. Arthropod Plant Interact..

[51] Trotter A. (1899). Credette Redi davvero che le galle ed i produttori di esse fossero generati da un’anima vegetativa delle piante?. Boll. Soc. Veneto-Trent. Sci. Nat..

[52] Trotter A. (1910). Le cognizioni cecidologiche e teratologiche di Ulisse Aldrovandi e della sua Scuola. Marcellia.

[53] Bertoloni Meli D. (2011). Mechanism, Experiment, Disease: Marcello Malpighi and Seventeenth-Century Anatomy.

[54] Santini L., Tongiorgi Tomasi L., Tongiorgi P. (1981). Francesco Redi e il problema delle galle: Un manoscritto inedito e la relativa iconografia. Redia.

[55] Generali D., Generali D. (2019). Un fronte della battaglia contro la tesi della generazione spontanea. Gli studi di Antonio Vallisneri sull’origine degli insetti delle galle e di altri parassiti di piante e animali. Ex ovo omnia. Parassitologia e Origine delle Epidemie nelle Ricerche e Nell’opera di Antonio Vallisneri.

[56] Favino F., Beretta M., Clericuzio A., Principe L.M. (2009). On the Cimento’s oak academies: An unknown contribution by Antonio Oliva. The Accademia del Cimento and Its European Context.

